# Editorial for the Special Issue on Wide Bandgap Semiconductor Based Micro/Nano Devices

**DOI:** 10.3390/mi10030213

**Published:** 2019-03-26

**Authors:** Jung-Hun Seo

**Affiliations:** Department of Materials Design and Innovation, University at Buffalo, The State University of New York, Buffalo, NY 14260, USA; junghuns@buffalo.edu

While conventional group IV or III-V based device technologies have reached their technical limitations (e.g., limited detection wavelength range or low power handling capability), wide bandgap (WBG) semiconductors which have band-gaps greater than 3 eV have gained significant attention in recent years as a key semiconductor material in high-performance optoelectronic and electronic devices [1,2]. These WBG semiconductors have various definitive advantages for optoelectronic and electronic applications due to their large bandgap energy. WBG energy is suitable to absorb or emit ultraviolet (UV) light in optoelectronic devices [3]. It also provides a higher electric breakdown field, which allows electronic devices to possess higher breakdown voltages [4].

In this Special Issue, 13 papers published, including various AlGaN/GaN, SiC, and WO_3_ based devices. More than half of papers reported recent progress on AlGaN/GaN high electron mobility transistors (HEMTs) and light emitting diodes (LEDs). Wojtasiak et al., and Sun et al, reported a structural modification of AlGaN/GaN HEMTs to improve turn-on voltage, contact resistance, and on-resistance [5]. Huang et al. investigated high-temperature characteristics of AlGaN/GaN HEMTs and successfully established the thermal model [6]. Mao et al. and Li et al. simulated AlGaN/GaN HEMTs with a large signal model to investigate the kink-effect [7,8]. All of these efforts toward AlGaN/GaN HEMTs enable readers to understand current issues in AlGaN/GaN HEMTs and offer various experimental and theoretical solutions. Beside transistor works, flip-chip GaN LEDs that were combined with TiO_2_/SiO_2_ distributed Bragg reflectors (DBRs) was reported by Zhou et al [9]. An improved GaN HEMTs and their microwave performance by employing the asymmetric power-combining was reported by Kim et al [10]. Along with another GaN LED built on a modified micron-size patterned sapphire substrate by Hsu et al. [11]. These GaN LED works are also guided broad readers in the field of optoelectronics and biomedical areas toward future high-performance optogenetics and photonics applications. Also, Sun et al. reported an enhanced AlGaN/GaN Schottky Barrier by engineering the structure of the diode [12].

In addition to AlxGa1-xN system, two SiC simulation efforts have been made by Huang et al. and Jia et al. Huang. They focused on the improvement of higher added efficiency (PAE) factor in 4H-SiC metal semiconductor field effect transistors and breakdown voltage of 4H-SiC diodes, respectively [13,14].

Besides popular AlxGa1-xN and SiC-based applications, three papers report InGaZnO thin-film transistors (TFTs), Si/GaP one-transistor dynamic random-access memory (1T DRAM), and WO_3_ thin-film. Zhou et al. investigated a stress tolerance of InGaZnO TFTs with a SiO_2_ or Al_2_O_3_ passivation layer which shows a stable positive bias during the operation [15]. Kim et al. simulated a novel 1T DRAM design by inserting a GaP pillar which showed a stable high-temperature operation [16]. Finally, Zhang et al. reported the changes of the optical bandgap in Tungsten trioxide by thermal annealing which can be used for various electrochromic devices [17].

To the end, I would like to take this opportunity to thank all the authors for submitting their papers to this special issue. I also want to thank all the reviewers for dedicating their time and helping to improve the quality of the submitted papers.

## References

[1] Kim M., Seo J.-H., Singisetti U., Ma Z. (2017). Recent advances in free-standing single crystalline wide band-gap semiconductors and their applications: GaN, SiC, ZnO, β-Ga_2_O_3_, and diamond. J. Mater. Chem. C.

[2] Swinnich E., Dave Y.J., Pitman E.B., Broderick S., Mazumder B., Seo J.-H. (2018). Prediction of optical band gap of β-(Al_x_Ga_1-x_)_2_O_3_ using material informatics. Mater. Discov..

[3] Liu D., Cho S.J., Park J., Gong J., Seo J.-H., Dalmau R., Zhao D., Kim K., Kim M., Kalapala A.R.K. (2018). 226 nm AlGaN/AlN UV LEDs using p-type Si for hole injection and UV reflection. Appl. Phys. Lett..

[4] Swinnich E., Hasan M.N., Zeng K., Dove Y., Singisetti U., Mazumder B., Seo J.-H. (2019). Flexible β-Ga_2_O_3_ Nanomembrane Schottky Barrier Diodes. Adv. Electron. Mater..

[5] Wojtasiak W., Góralczyk M., Gryglewski D., Zając M., Kucharski R., Prystawko P., Piotrowska A., Ekielski M., Kamińska E., Taube A. (2018). AlGaN/GaN High Electron Mobility Transistors on Semi-Insulating Ammono-GaN Substrates with Regrown Ohmic Contacts. Micromachines.

[6] Huang H., Li F., Sun Z., Cao Y. (2018). Model Development for Threshold Voltage Stability Dependent on High Temperature Operations in Wide-Bandgap GaN-Based HEMT Power Devices. Micromachines.

[7] Mao S., Xu Y. (2018). Investigation on the I–V Kink Effect in Large Signal Modeling of AlGaN/GaN HEMTs. Micromachines.

[8] Li J., Mao S., Xu Y., Zhao X., Wang W., Guo F., Zhang Q., Wu Y., Zhang B., Chen T. (2018). An Improved Large Signal Model for 0.1 μm AlGaN/GaN High Electron Mobility Transistors (HEMTs) Process and Its Applications in Practical Monolithic Microwave Integrated Circuit (MMIC) Design in W band. Micromachines.

[9] Zhou S., Xu H., Liu M., Liu X., Zhao J., Li N., Liu S. (2018). Effect of Dielectric Distributed Bragg Reflector on Electrical and Optical Properties of GaN-Based Flip-Chip Light-Emitting Diodes. Micromachines.

[10] Kim S., Lee M.-P., Hong S.-J., Kim D.-W. (2018). Ku-Band 50 W GaN HEMT Power Amplifier Using Asymmetric Power Combining of Transistor Cells. Micromachines.

[11] Hsu W.-Y., Lian Y.-C., Wu P.-Y., Yong W.-M., Sheu J.-K., Lin K.-L., Wu Y.S. (2018). Suppressing the initial growth of sidewall GaN by modifying micron-sized patterned sapphire substrate with H_3_PO_4_-based etchant. Micromachines.

[12] Sun Y., Wang Y., Tang J., Wang W., Huang Y., Kuang X. (2019). A Breakdown Enhanced AlGaN/GaN Schottky Barrier Diode with the T-Anode Position Deep into the Bottom Buffer Layer. Micromachines.

[13] Huang Y., Wang Y., Kuang X., Wang W., Tang J., Sun Y. (2018). Step-Double-Zone-JTE for SiC Devices with Increased Tolerance to JTE Dose and Surface Charges. Micromachines.

[14] Jia H., Hu M., Zhu S. (2018). An Improved UU-MESFET with High Power Added Efficiency. Micromachines.

[15] Zhou Y., Dong C. (2018). Influence of Passivation Layers on Positive Gate Bias-Stress Stability of Amorphous InGaZnO Thin-Film Transistors. Micromachines.

[16] Kim M., Ha J., Kwon I., Han J.-H., Cho S., Cho I. (2018). A Novel One-Transistor Dynamic Random-Access Memory (1T DRAM) Featuring Partially Inserted Wide-Bandgap Double Barriers for High-Temperature Applications. Micromachines.

[17] Zhang G., Lu K., Zhang X., Yuan W., Shi M., Ning H., Tao R., Liu X., Yao R., Peng J. (2018). Effects of Annealing Temperature on Optical Band Gap of Sol-gel Tungsten Trioxide Films. Micromachines.

